# The Effect of Optimally Timed Osteopathic Manipulative Treatment on Length of Hospital Stay in Moderate and Late Preterm Infants: Results from a RCT

**DOI:** 10.1155/2014/243539

**Published:** 2014-11-25

**Authors:** Gianfranco Pizzolorusso, Francesco Cerritelli, Alessandro Accorsi, Chiara Lucci, Lucia Tubaldi, Jenny Lancellotti, Gina Barlafante, Cinzia Renzetti, Carmine D'Incecco, Francesco Paolo Perri

**Affiliations:** ^1^European Institute for Evidence Based Osteopathic Medicine (EBOM), Viale Unità d'Italia 1, 66100 Chieti, Italy; ^2^Accademia Italiana Osteopatia Tradizionale (AIOT), Via Prati 29, 65124 Pescara, Italy; ^3^Neonatal Intensive Care Unit, Macerata Public Hospital, Via Santa Lucia 2, 62100 Macerata, Italy; ^4^Neonatal Intensive Care Unit, Pescara Public Hospital, Via Fonte Romana, 65124 Pescara, Italy

## Abstract

*Introduction*. Little research has been conducted looking at the effects of osteopathic manipulative treatment (OMT) on preterm infants. *Aim of the Study*. This study hypothesized that osteopathic care is effective in reducing length of hospital stay and that early OMT produces the most pronounced benefit, compared to moderately early and late OMT. A secondary outcome was to estimate hospital cost savings by the use of OMT. *Methods*. 110 newborns ranging from 32- to 37-week gestation were randomized to receive either OMT or usual pediatric care. Early, moderately early, and late OMT were defined as <4, <9, and <14 days from birth, respectively. *Result*. Hospital stay was shorter in infants receiving late OMT (−2.03; 95% CI −3.15, −0.91; *P* < 0.01) than controls. Subgroup analysis of infants receiving early and moderately early OMT resulted in shorter LOS (early OMT: −4.16; −6.05, −2.27; *P* < 0.001; moderately early OMT: −3.12; −4.36, −1.89; *P* < 0.001). Costs analysis showed that OMT significantly produced a net saving of €740 (−1309.54, −170.33; *P* = 0.01) per newborn per LOS. *Conclusions*. This study shows evidence that the sooner OMT is provided, the shorter their hospital stay is. There is also a positive association of OMT with overall reduction in cost of care.

## 1. Introduction

Preterm birth, defined by the American Academy of Pediatrics and the World Health Organization as birth, that occurs in or before the end of the 37th week of pregnancy [1, 2] represents a substantial problem in perinatal medicine worldwide [3].

Moderate preterm (MP) and late preterm (LP) infants have been loosely classified by current literature [4–6], as comprising the greatest proportion of the preterm population [7]. Extensive reviews [8, 9] address the current epidemiology, care, and outcomes of LP infants, documenting the overall impact on health care services [10].

Although length of stay (LOS) for MP and LP infants is much less than that for extremely preterm infants, the impact in terms of hospital stays is similar because of the large volume of these two groups [11, 12].

Preterm birth has been also associated with a negative socioeconomic impact. Mean hospital costs associated with LOS are approximately between US$1,000 for a term infant and US$40,000 for an infant born either moderately or late preterm [13].

Noninvasive treatment aimed at reducing LOS of preterm infants and cost of neonatal care could well prove an appealing option worth investigating.

Very little research has been conducted looking at the effects of osteopathic manipulative treatment (OMT) on preterm infants [14–16]. In the setting of the NICU, data suggests that OMT improves gastrointestinal function [15], reduces hospital stay [14, 15], and enhances nipple feeding function [16].

However, the application of OMT in preterm infants remains uncertain and a more rigorous study design is warranted.

The objective of the present randomized controlled trial (RCT) was to investigate the effectiveness of OMT in reducing hospital stay and related costs in MP and LP infants.

## 2. Osteopathic Care

Osteopathic medicine is a form of drug-free noninvasive manual medicine. It relies on manual contact for diagnosis and treatment. Osteopathic practitioners use a wide variety of therapeutic manual techniques [20] to improve physiological function and support homeostasis that has been altered by somatic dysfunction.

In the osteopathic literature, somatic dysfunction is defined as “impaired or altered function of the somatic (body framework) system: skeletal, arthrodial and myofascial structures and their related vascular, lymphatic and neural elements” [17].

Two essential components of osteopathic health care are the structural evaluation of the patient for somatic dysfunction diagnosis and an array of manipulative techniques for treatment [21].

The aim of a structural examination is to locate the somatic dysfunction. In infants, the structural exam is usually performed with the patient lying down. Diagnostic criteria for somatic dysfunction are focused on tissue texture abnormalities and tone. Areas of asymmetry and misalignment of bony landmarks are evaluated. The quality of motion, its balance, and organization are noted.

In treating preterm infants during the very first days of life, osteopaths use several manual techniques [15, 22], in order to ameliorate diagnosed SD and promote health both in the presence and in the absence of obvious disease [23, 24].

## 3. Materials and Methods

### 3.1. Aim of the Study

This study hypothesized that osteopathic care is effective in reducing the length of hospital stay in a population of MP and LP infants and that early OMT produces the most pronounced benefit (primary outcome). Secondary outcome was to estimate the potential savings, in terms of hospital costs related to LOS, by the use of OMT.

### 3.2. Study Population

MP and LP infants entering the NICU of Macerata Public Hospital from October 2010 to July 2012 were assessed for eligibility. All subjects met the following criteria: infant born at Macerata Public Hospital with a gestational age between 32 and 37 weeks, preterm infant free of medical complications and with written informed consent from parents or legal guardians. Exclusion criteria, applied at study entry and to any portion of the infant's hospital course, included the following: gestational age <32 or >37 weeks, first OMT provided after 14 days from birth; genetic/congenital disorders; cardiovascular abnormalities; proven or suspected necrotized enterocolitis with or without gastrointestinal perforation; proven or suspected abdominal obstruction; pre/postsurgery patients; pneumoperitoneum; newborns from an HIV seropositive or drug addicted mother; infants transferred to or from another unit or hospital; early postnatal discharge (defined as a hospital stay of <48 hours after delivery).

The trial was approved by the Ethics Committee of Macerata Public Hospital (number 22/int./CEI/27239) and was registered at ClinicalTrials.gov (Registration Number: NCT01784835).

### 3.3. Osteopaths in Charge of the Study

4 osteopaths certified by the Registro degli Osteopati d'Italia were involved and randomly divided into two groups: 2 osteopaths performed the structural evaluation (group A) and 2 osteopaths performed the structural evaluation and the treatment (group B).

In order to provide blinding and avoid possible confounding, osteopaths from groups A and B were allowed to enter the NICU two days per week and during different hours.

None of the osteopathic practitioners in either group were involved in the study design, data entry, or statistical analysis.

### 3.4. Study Group

Newborns allocated to the study group received standard pediatric care plus two osteopathic treatments per week, for the whole hospitalization period.

OMT included the application of a selected range of manipulative techniques (Table 1), depending on the findings from the structural examination of the infant.

Each OMT session lasted 20 minutes and was not based on a predetermined protocol and the treatment was not standardized. Data on structural examinations and OMT techniques are not reported as this was not the focus of this study.

### 3.5. Control Group

Preterm infants in the control group received standard pediatric care plus two osteopathic structural evaluations per week. The structural evaluations were performed according to the same schedule as the study group and lasted 10 minutes. Once completed, osteopaths from group A remained standing in front of the incubators or open cribs for an additional 10 minutes so that the total time spent with each infant in both the control and treatment groups was equal, to further assist in blinding the ancillary NICU staff.

### 3.6. Allocation Concealment and Blinding

Subjects were randomly allocated to study or control group by an information technology consultant. A permuted-block randomization with an allocation ratio of 1 : 1 was performed using the R software as computer random generator [25].

NICU staff were blinded to outcome measurements and patient group assignment.

### 3.7. Data Collection

Data was collected using EBOM-GCCN software [14]. Nursing and medical records were collected daily by the NICU staff, from infant birth until the time of discharge.

Maternal data was also obtained and included reported pregnancy complications, single versus multiple gestation, fetal presentation, type of delivery, premature rupture of membranes, and abruption of the placenta.

Neonatal data collected included gender, gestational age, infants small for gestational age, birth weight, neonatal complications (diagnosed at birth and during hospitalization), and diagnosis-related group (DRG).

### 3.8. Statistical Analyses

Sample size calculation used multiple regression method. An effect size of 0.3 was applied and a total number of predictors equal to 4 were considered. Defining a statistical power of 0.90 and an alpha level equal to 0.05, a sample size of 52 subjects per group was computed. To allow for attrition, the sample size was increased to 55 subjects per group.

The assessment of normality was performed using Shapiro's test.

Statistical analyses were based on intention-to-treat model. Missing data was handled using last observation carried forward imputation technique. The baseline characteristics of the population were analyzed using descriptive statistics. Univariate statistical tests were used to compare the study and control groups at baseline.

A generalized linear model was performed to study the independent effect of OMT on LOS, taking into account the following confounders: gender, gestational age, birth weight, DRG-386 (prematurity with respiratory distress syndrome), DRG-387 (prematurity with major complications), and DRG-388 (prematurity without major complications) [26].

A subgroup analysis was performed to address the relationship between the timing of the first OMT and LOS.

Depending on when the first OMT was provided, three different time frames were established as follows:

- early OMT as <4 days from birth;
- moderately early OMT as <9 days from birth;
- late OMT as <14 days from birth.

The use of three different time frames resulted from the osteopathic care schedule. Osteopathic care was provided 2 days per week on Mondays and Thursdays.

The statistical program used for data analyses was R (version 2.15.0) [25].

### 3.9. Cost Analysis

A multivariate analysis was performed to study the average hospitalization costs among infants of study and control groups. Cost data was extracted from 2011 administrative databases of the Regional Office of the Ministry of Health (ROMH) of Marche, where the NICU of the present RCT is located. More specifically, the Ministry of Health, besides its central offices, is divided into regional offices distributed throughout Italy. In relation to their individual skill sets, each ROMH carries out activities of control and offers services to the population. Then, the Istituto Superiore di Sanità (National Healthcare Institute) estimates a precise amount of reimbursement for each DRG and hospitals receive funds according to patients DRG.

In this trial, DRG and reimbursements considered were DRG-386, prematurity with respiratory distress syndrome (12.932,69€), DRG-387, prematurity with major complications (7.450,09€), and DRG-388, prematurity without major complications (3.757,22€) [26].

For this study, the cost of each OMT was theoretically set at 20,00€ [27], taking into account the guidelines from Fondo Assistenza Sanitaria Dirigenti Aziende Commerciali (FASDAC), an Italian private-sector health insurance fund for managers and CEOs.

Ordinary least squares regression was used to investigate the effects of OMT on hospitalization costs after adjusting for gender, gestational age, LOS, and birth weight.

Cost estimates were adjusted for inflation to 2012 euros using the Medical Component of the Consumer Price Index.

## 4. Results

**Figure 1:**
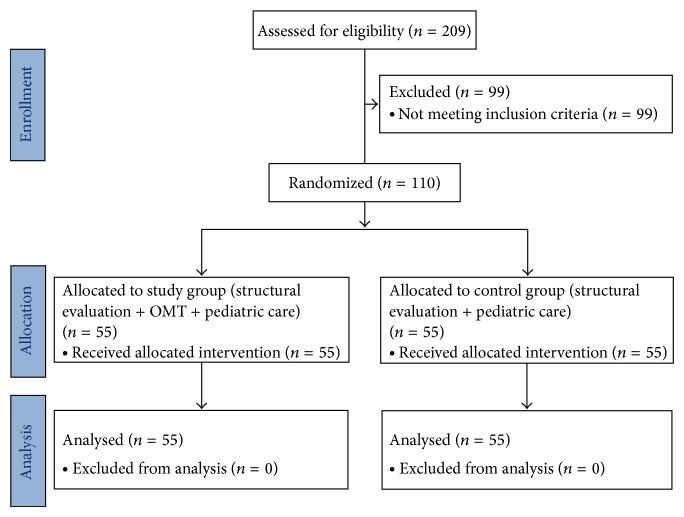
Flowchart of the study.

As shown in Figure 1, 209 newborns entered the study and were assessed for eligibility.

After the application of exclusion criteria, *N* = 110 were selected for the final sample and randomized 1 : 1 to study group (*N* = 55) and control group (*N* = 55).

None of the subjects dropped out up during the trial and no adverse events were recorded.

The assessment of normality for gestational age (*W* = 0.95, *P* = 0.65) and birth weight (*W* = 0.97, *P* = 0.47) resulted in a Gaussian distribution.

As shown in Table 2, neonatal and maternal characteristics at baseline were similar across the two groups, except for a higher number of vaginal delivery in the control (*P* = 0.03).

**Figure 2:**
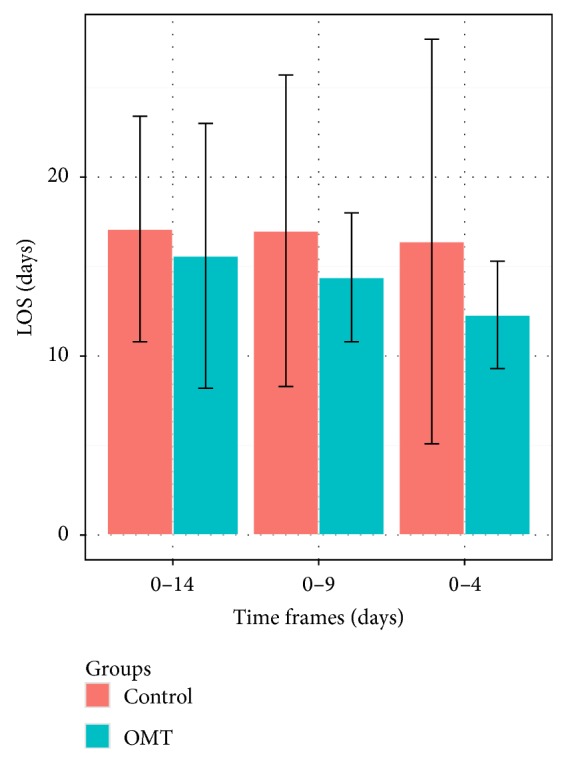
Mean LOS (days ± SD) differences between study and control groups according to OMT time frames.

The mean LOS was 15.6 ± 7.4 for the study group and 17.1 ± 6.3 for the control group (*P* < 0.05) (Figure 2).

The first multivariate linear model was run to test the independent effect of factors associated with LOS.

**Figure 3:**
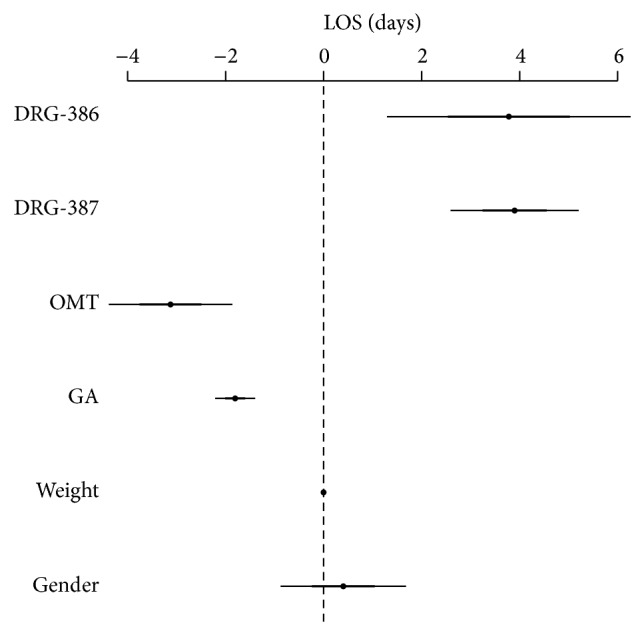
Generalized linear model for LOS with late OMT (time frame <14 days; *N* = 55). DRG-386 = diagnosis related groups, prematurity with respiratory distress syndrome; DRG-387 = diagnosis related groups, prematurity with major complications; OMT = osteopathic manipulative treatment; GA = gestational age.

As shown in Figure 3, late OMT (<14 days from birth) and gestational age were negatively associated with LOS. More specifically, being exposed to late OMT resulted in an earlier discharge (−2.03; 95% CI −3.15, −0.91; *P* < 0.01), as well as one-week increases in gestational age reduced LOS (−1.75; −2.11, −1.38; *P* < 0.001). Results also showed that both DRG-387 and -386 significantly increased hospitalization by almost 4 days (3.94; 2.76–5.11; *P* < 0.001 and 3.75; 1.67–5.82; *P* < 0.001, resp.).

### 4.1. Subgroup Analysis according to OMT Time Frames

A subgroup analysis was performed to investigate the relationship among early OMT, moderately early OMT, and hospital stay.

#### 4.1.1. Moderately Early OMT Group

Using the time frame of 9 days (moderately early OMT), the initial sample was reduced to 91 subjects. Among them, 43 were in the study group and 47 were controls.

At baseline, the two groups were well matched for neonatal and maternal characteristics (Table 3). At the end of the study, univariate analysis (Figure 2) showed mean LOS being 14.4 ± 3.6 days for infants in the study group and 17.0 ± 8.7 for controls (*P* < 0.01).

**Figure 4:**
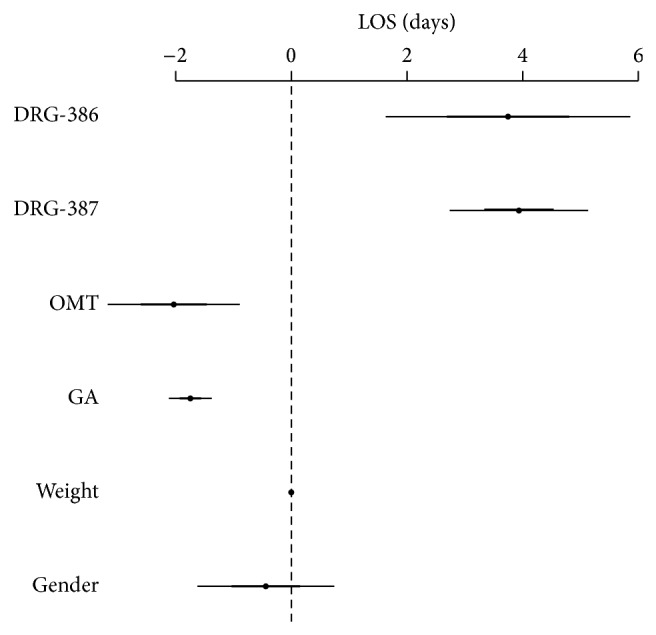
Generalized linear model for LOS with moderately early OMT time frame (<9 days; *N* = 43). DRG-386 = diagnosis related groups, prematurity with respiratory distress syndrome; DRG-387 = diagnosis related groups, prematurity with major complications; OMT = osteopathic manipulative treatment; GA = gestational age.

A second linear regression model was run to study the independent effect of moderately early OMT (<9 days from birth) on hospital stay (Figure 4). Results showed the following significant association with decreased LOS: OMT (−3.12; −4.36, −1.89; *P* < 0.001); gestational age (−1.81; −2.21, −1.40; *P* < 0.001), DRG-387 (3.90; 2.61, 5.18; *P* < 0.001), DRG-386 (3.78; 1.33, 6.23; *P* < 0.01); birth weight (−0.002; −0.004, −0.0002; *P* < 0.05).

#### 4.1.2. Early OMT Group

In looking at those subjects receiving early OMT (<4 days), the sample was further reduced to 55 subjects, 26 in the study group and 29 controls. At baseline, study and control groups were balanced with regard to neonatal and maternal characteristics (Table 4). At discharge (Figure 2), mean LOS was 12.3 ± 3.0 days for preterm infants in the study group and 16.4 ± 11.3 for those in the control group (*P* < 0.001).

**Figure 5:**
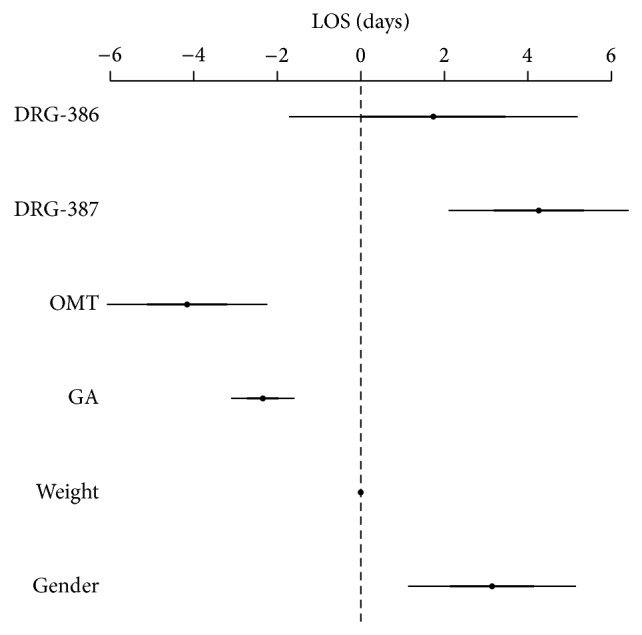
Generalized linear model for LOS with early OMT (time frame <4 days; *N* = 26). DRG-386 = diagnosis related groups, prematurity with respiratory distress syndrome; DRG-387 = diagnosis related groups, prematurity with major complications; OMT = osteopathic manipulative treatment; GA = gestational age.

A third linear regression model was performed to measure the independent effect of early OMT (<4 days from birth) on hospitalization (Figure 5). The following associations with LOS were found: OMT (−4.16; −6.05, −2.27; *P* < 0.001); gestational age (−2.35; −3.09, −1.60; *P* < 0.001); gender (3.14; 1.16, 5.12; *P* < 0.01); birth weight (−0.003; −0.005, −0.0005; *P* < 0.05); DRG-387 (4.26; 2.13, 6.39; *P* < 0.01); DRG-386 (1.74; −1.67, 5.14; *P* < 0.32).

#### 4.1.3. Cost Analysis

As far as cost analysis is concerned, in the present study OMT represented a cost-saving procedure. Although results from univariate statistical analysis showed that mean costs for study group and controls did not vary significantly (5324 ± 1634 versus 5499 ± 2681, resp., *P* = 0.68), the use of OMT produced a net saving of approximately 175,00€ (95% CI −671, 1020) per newborn per LOS, providing an overall cost reduction of almost 10.000€ (−36905, 56100).

The ordinary least square regression took into account several potential confounders listed as follows: gender, gestational age, birth weight, LOS, OMT time frame, OMT, DRG-388, DRG-387, and DRG-386. Results were expressed in terms of mean cost savings between each confounder and DRG-388 was set as the reference category. As shown in Table 5, OMT significantly produced a net saving of €740 (−1309,54, −170,33; *P* = 0.01) per newborn. Results also confirmed cost increases according to DRG-387 (€1883,12; 1275,93, 2490,31; *P* < 0.001), DRG-386 (€5190,54; 4099,19, 6281,89; *P* < 0.001), and OMT time frame (€102,09; 14,30, 189,87; *P* = 0.02).

## 5. Discussion

The present study was designed to yield accurate quantitative data on the effectiveness of OMT in preterm infants, especially those born either moderately or later preterm.

The major findings in this population-based study of length of hospital stay in MP and LP infants were that OMT is effective in reducing LOS and the sooner treatment was received the sooner infants were discharged from hospital.

In detail, a mean reduction of 2 days was found to be statistically significant in infants receiving late OMT (within 14 days from birth) compared with controls (95% CI −3.15, −0.91; *P* < 0.01). Subgroup analysis of infants under early (before day 4) and moderately early OMT (before day 9) showed a linear correlation between LOS and time frame of first OMT. It was found that early OMT was associated with the shortest LOS (−4.16 days; −6.05, −2.27; *P* < 0.001) and moderately early OMT produced a reduced hospital stay of 3 days (−3.12; −4.36, −1.89; *P* < 0.001).

Although OMT has been barely studied in the care of premature infants [14–16] our findings are consistent with the results of the exploratory study by Pizzolorusso and the recently published trial by Cerritelli. LOS reduction in the RCT by Cerritelli et al. [14] was higher than that found in the present trial (−5.91; −7.94, −3.87; *P* < 0.001 versus −2.03; −3.15, −0.91; *P* < 0.01), but this difference is smaller in the group of infants who received early OMT (−5.91; −7.94, −3.87; *P* < 0.001 versus −4.16; −6.05, −2.27; *P* < 0.001) and might be related to the different characteristics of the samples under study. On the other hand, data by Pizzolorusso et al. [15] do not compare with these results due to the application of multivariate logistic regression run to obtain risk adjusted estimates of odds ratios.

Finally, the potential economic benefits of the use of OMT in NICU were investigated. In the present study, OMT produced a cost saving per infant of €740,00, in contrast with €3000,00 per infant found by Cerritelli et al. [14]. Results from the ordinary least square regression showed also that the time frame of the first OMT played an important role in terms of an additional cost saving of €102,00 per day.

Possible explanations to the positive effects of OMT in the reduction of hospital stay are difficult to outline although several mechanisms are proposed.

### 5.1. OMT Improves Feeding Difficulty in Preterm Infants

Feeding difficulty in preterm infants is a major cause of delayed discharge [4].

In 2011, Lund described a case of hospitalized premature twins with nipple-feeding dysfunction. After the application of OMT, nipple-feeding performance improved to full oral feeding, suggesting the positive effect of OMT in the coordination of suck and swallowing.

In the same year, Pizzolorusso et al. investigated the effects of OMT in 350 premature infants. The data suggested that patients from the study group experienced fewer gastrointestinal symptoms such as vomiting, regurgitation, and gastric residuals.

### 5.2. OMT Improves Autonomic Function in Infants and Adults

In MP and LP infants, the autonomic nervous system undergoes significant maturation between 31 and 38 weeks of gestation, represented by decreases in heart rate and increases in heart rate variability (HRV) [28]. In light of the available osteopathic literature [29–31], authors speculate that OMT may also affect autonomic function and HRV in preterm infants, enhancing the balance between sympathetic and parasympathetic systems.

### 5.3. OMT Plays an Important Role in Inflammation

In 2007 Meltzer and Standley [32] developed an in vitro model of human fibroblasts and performed experiments on strain-regulated fibroblast-derived cytokines. They concluded that OMT techniques modulate the secretion of proinflammatory and anti-inflammatory interleukins.

Given the evidence that the skin surface of neonates ≤32 weeks GA have higher levels of proinflammatory cytokines than full term infants [33], the authors hypothesize that OMT applied in preterm infants may improve inflammatory and neuroendocrine responses.

### 5.4. Limitations

One of the limitations of this study is that it was conducted in only one NICU located in Central Italy. Potential differences in practice may exist in other institutions, influencing generalizability of our findings.

Furthermore, discharge plan for study and control groups only took into account the health of the child and followed the proposed guidelines of the AAP [34]. Parental planning was not addressed during the trial, as well as feeding skills of the mother and the availability of support at home.

Finally, cost estimates were based on a theoretical approach based on mathematical computation of hospital net savings, rather than the real hospital stay.

## 6. Conclusions

The present RCT shows evidence that osteopathic care in NICU offers advantages in the reduction of hospital stay.

The approach taken, focusing on the impact of different time frames (early, moderately early, and late) of first OMT, is rather novel and well thought out for substantiating the results.

Our findings may be of interest and use to researchers, stakeholders, and policy-makers planning to evaluate the impact of osteopathic care in national health care systems, particularly those wishing to incorporate within a decision analytic framework the economic impact of MP and LP birth and the cost-effectiveness of treatment strategies.

RCTs based on multicentric design are warranted before any firm conclusions can be drawn.

## Figures and Tables

**Table 1:** Descriptions of the osteopathic manipulative techniques used in the study.

Indirect myofascial release [17]	The osteopath moves the dysfunctional tissues away from the restrictive barrier (a functional limit that abnormally diminishes the normal physiologic range of motion) until tissue tension is equal in one or all planes and directions.

Balanced ligamentous tension [17, 18]	According to Dr. Sutherland's model, all the joints in the body are balanced ligamentous articular mechanisms. To release articular strain, the osteopath seeks to position the bones of a joint at the point of balanced ligamentous tension.

Balanced membranous tension [19]	The osteopath uses balanced membranous tension techniques to normalize the articular dysfunctions of the cranium, face, and sacrum that involves the dura mater. The goal of treatment is to position the bones making up the articulation at the point of balanced membranous tension.

**Table 2:** General characteristics of the study population at baseline with OMT time frame <14 days.

	Study group (*n* = 55)	Control group (*n* = 55)	*P* value
Neonatal			
Males^*^	27 (49.1)	25 (45.5)	0.70
Gestational age (w)	33.8 (2.0)	34.3 (1.6)	0.13
Birth weight (gr.)	2144 (556)	2226 (463)	0.40
Small for gestational age^*^	9 (16.4)	9 (16.4)	1.00
Complications^§^			
Jaundice^*^	18 (32.7)	16 (29.1)	0.73
Feeding^*^	14 (25.5)	9 (16.4)	0.30
Esophageal reflux^**^	2 (3.6)	1 (1.8)	0.56
Respiratory^*^	12 (21.8)	7 (12.7)	0.25
Endocrine and metabolic^*^	12 (21.8)	18 (32.7)	0.27
DRG^*^			0.26
386	4 (7.3)	4 (7.3)	
387	23 (41.8)	15 (27.3)	
388	28 (50.9)	36 (65.4)	
Maternal			
Total number of women	46	51	0.61
Single gestation^*^	37 (80.4)	47 (92.2)	0.28
Multiple gestation^*^	9 (19.6)	4 (7.8)	0.17
Vaginal delivery^*^	11 (23.9)	24 (47.1)	0.03
C section^*^	35 (76.1)	27 (52.9)	0.31
Cephalic presentation^*^	54 (98.1)	53 (96.4)	0.92
Breech presentation^**^	1 (1.9)	2 (3.6)	0.56
Pregnancy^§§^			
No complications^*^	29 (78.4)	38 (80.6)	0.27
Gestational diabetes^**^	4 (10.8)	3 (6.5)	0.71
Infections^**^	1 (2.7)	2 (4.3)	0.56
Other conditions^**^	0 (0.0)	2 (4.3)	0.16
Placenta abruption^**^	1 (2.7)	0 (0.0)	0.32
PROM^**^	2 (5.5)	2 (4.3)	1.00

DRG: diagnosis related groups. Numbers are mean (SD). *P* value from *t*-test. ^*^ *n* (%), *P* value from Fisher's test. ^§^Complications were classified according to ICD-9 codes. ^§§^Pregnancy data were classified according to ICD-9 diagnosis codes. ^**^ *n* (%), *P* value from Fisher's exact test.

**Table 3:** General characteristics of the study population at baseline with OMT time frame <9 days.

	Study group (*n* = 43)	Control group (*n* = 47)	*P* value
Neonatal			
Males^*^	21 (48.8)	26 (55.3)	0.69
Gestational age (w)	33.9 (2.1)	34.4 (1.6)	0.22
Birth weight (gr.)	2206 (605)	2282 (466)	0.51
Small for gestational age^*^	5 (11.6)	6 (12.8)	0.76
Complications^§^			
Jaundice^*^	15 (34.9)	14 (29.8)	0.85
Feeding^*^	9 (20.9)	7 (14.9)	0.62
Esophageal reflux^**^	1 (2.3)	1 (2.1)	1.00
Respiratory^*^	9 (20.9)	12 (25.5)	0.51
Endocrine & metabolic^*^	13 (30.2)	15 (31.9)	0.71
DRG^*^			0.60
386	3 (7.0)	3 (6.3)	
387	16 (37.2)	13 (27.7)	
388	24 (55.8)	31 (66.0)	
Maternal			
Total number of women	37	44	0.44
Single gestation^*^	30 (81.1)	42 (95.5)	0.16
Multiple gestation^*^	7 (18.9)	2 (4.5)	0.10
Vaginal delivery^*^	10 (27.0)	12 (27.3)	0.67
C section^*^	27 (73.0)	32 (72.7)	0.51
Cephalic presentation^*^	42 (97.7)	45 (95.7)	0.75
Breech presentation^**^	1 (2.3)	2 (4.3)	0.56
Pregnancy^§§^			
No complications^*^	20 (66.7)	19 (57.7)	0.87
Gestational diabetes^**^	4 (13.3)	3 (9.1)	0.71
Infections^**^	1 (3.3)	2 (6.1)	0.56
Other conditions^**^	4 (13.4)	5 (15.2)	0.74
Placenta abruption^**^	0 (0.0)	1 (3.0)	0.32
PROM^**^	1 (3.3)	1 (3.0)	1.00

DRG: diagnosis related groups. Numbers are mean (SD). *P* value from *t*-test. ^*^ *n* (%), *P* value from Fisher's test. ^§^Complications were classified according to ICD-9 codes. ^§§^Pregnancy data were classified according to ICD-9 diagnosis codes. ^**^ *n* (%), *P* value from Fisher's exact test.

**Table 4:** General characteristics of the study population at baseline with OMT time frame <4 days.

	Study group (*n* = 26)	Control group (*n* = 29)	*P* value
Neonatal			
Males^*^	14 (53.9)	15 (51.7)	1.00
Gestational age (w)	34.7 (1.7)	35.0 (1.2)	0.46
Birth weight (gr.)	2402 (592)	2395 (493)	0.96
Small for gestational age^*^	5 (19.2)	3 (10.3)	0.48
Complications^§^			
Jaundice^*^	8 (30.8)	9 (31.0)	0.81
Feeding^*^	3 (11.5)	1 (3.4)	0.32
Esophageal reflux^**^	0 (0.0)	1 (3.4)	0.32
Respiratory^*^	6 (23.1)	8 (27.6)	0.59
Endocrine and metabolic^*^	9 (34.6)	10 (34.5)	0.82
DRG^*^			0.51
386	1 (3.9)	3 (10.3)	
387	9 (34.6)	7 (24.1)	
388	16 (61.5)	19 (65.5)	
Maternal			
Total number of women	23	27	0.57
Single gestation^*^	19 (82.6)	26 (96.3)	0.30
Multiple gestation^*^	4 (17.4)	1 (3.7)	0.18
Vaginal delivery^*^	7 (30.4)	10 (37.0)	0.47
C section^*^	16 (69.6)	17 (63.0)	0.86
Cephalic presentation^*^	26 (100.0)	24 (96.0)	0.78
Breech presentation^**^	0 (0.0)	1 (4.0)	0.32
Pregnancy^§§^			
No complications^*^	13 (68.4)	19 (76.0)	0.29
Gestational diabetes^**^	2 (10.5)	2 (8.0)	1.00
Infections^**^	0 (0.0)	1 (4.0)	0.32
Other conditions^**^	3 (15.8)	3 (12.0)	1.00
Placenta abruptio^**^	0 (0.0)	0 (0.0)	1.00
PROM^**^	1 (5.3)	0 (0.0)	0.32

DRG: diagnosis related groups. Numbers are mean (SD). *P* value from *t*-test. ^*^ *n* (%), *P* value from Fisher's test. ^§^Complications were classified according to ICD-9 codes. ^§§^Pregnancy data were classified according to ICD-9 diagnosis codes. ^**^ *n* (%), *P* value from Fisher's exact test.

**Table 5:** Results of ordinary least square regression for cost estimates.

	Costs (2012€)		
	Estimate	95% CI	*P* value
Gender	375.67	−208.89–960.24	0.21
Gestational age	159.93	−46.45–366.31	0.12
Birth weight (gr.)	−0.62	−1.36–0.12	0.10
LOS	62.66	10.68–114.64	0.02
OMT time frame	102.09	14.30–189.87	0.02
OMT	−739.94	−1309.54–−170.33	0.01
DRG-388 (R.C.)	1	1	1
DRG-387	1883.12	1275.93–2490.31	<0.001
DRG-386	5190.54	4099.19–6281.89	<0.001

LOS = length of stay; OMT = osteopathic manipulative treatment; R.C. = reference category. DRG-388 = diagnosis related groups, prematurity without major complications; DRG-387 = diagnosis related groups, prematurity with major complications; DRG-386 = diagnosis related groups, prematurity with respiratory distress syndrome.
